# Prevalence and associated factors of neonatal mortality in Ethiopia

**DOI:** 10.1038/s41598-022-16461-3

**Published:** 2022-07-15

**Authors:** Walelgn Gete Alamirew, Denekew Bitew Belay, Melkamu A. Zeru, Muluwerk Ayele Derebe, Senait Cherie Adegeh

**Affiliations:** Department of Statistics, College of Science, Bahir Dar University, Bahir Dar, Ethiopia

**Keywords:** Health care, Risk factors

## Abstract

Neonatal mortality is the death of a live-born baby within the first 28 days of birth. For the selected households, neonatal mortality was collected from children aged 0–28 days and women aged 15–49. The neonatal period is a significant 4-week period in human life because it carries a greater mortality risk. To identify the determinant factors of neonatal mortality in Ethiopia based on EDHS 2016 data with the application of count regression models. In this study, all neonates in Ethiopia were born within the 5 years preceding EDHS 2016 of the source population in the selected EAs from September to December 2015. Count regression models were used to analyze the data. A total of 10,641 live-born neonates within the previous 5 years of EDHS 2016 had neonatal mortality of women aged 15–49, which was considered in the study to be 7193. The data were found to have excess zeros (96.6%), and the variance (0.052) was higher than its mean (0.04). The count regression model (ZINB) was best fitted to the data with maximum likelihood parameter estimation methods. The average neonatal mortality difference in multiple births was increased by IRR = 8.53 times compared with a single birth. The average number of neonatal deaths experienced during breastfeeding was lower (IRR = 0.38) than that experienced by mothers who did not experience breastfeeding their child. The average neonatal mortality difference in rural residences was increased by IRR = 3.99 times compared to urban mothers' residences. In this study, the prevalence of Neonatal mortality in Ethiopia was higher. For selected ZINB count regression models of explanatory variables, such as multiple birth types, having rural residence factors of neonatal mortality increased the risk of death. However, having early breastfeeding, a female household head, and antenatal visits (1–4) and (5–10) during pregnancy decrease the risk of neonatal death.

## Introduction

Neonatal mortality is the death of a live-born baby within the first 28 days of birth. For the selected households, neonatal mortality was collected from children aged 0–28 days and women aged 15–49. The neonatal period is a significant 4-week period in human life because it carries a great risk of mortality^1^. In comparison, the probability of dying after the first month and before reaching age 1 was estimated at 11 deaths per 1000, and the probability of dying after reaching age 1 and before reaching age 5 was estimated at 10 deaths per 1000 in 2019^2^. The average daily mortality rate during the neonatal period is close to 30-fold higher than during the postnatal period (1 month to 1 year of age)^3^.

Globally, 4 million neonates die every year before they reach the age of 1 month^4^. Approximately two-thirds of all neonatal deaths worldwide occur in only 10 countries. Even though Ethiopia is among the countries with the largest percentage decline in the neonatal mortality rate from 1990 to 2013, 3% of global neonatal deaths (84,000 in 2013) occurred in the country, which places it among the top six countries, following India, China, Pakistan, Nigeria, and Bangladesh, with the highest number of neonatal deaths^4^. Moreover, the most recent growth reports on global trends in neonate mortality have shown alarmingly slow progress in curbing mortality rates among neonates, the slowest being in sub-Saharan Africa^5^.

The reduction in neonatal mortality rates has been slower than both under five and child mortality rates after the first month of life^6^. The good news is that neonatal mortality is on the decline globally. The world’s neonatal mortality rate fell from 33 deaths per 1000 live births in 1990 to 21 per 1000 in 2012. All regions showed drops, with lower percentage reductions in South Asia and sub-Saharan Africa (39% and 28%, respectively) than the other regions, even though the smaller regional reductions represent significant progress. The overall result was a reduction in global neonatal deaths from 4.6 million in 1990 to 2.9 million in 2012^7^.

The average neonatal mortality rate (NMR) in low-income countries (36/1000) was nine times higher than the average NMR in high-income countries (4/1000) and 1.5 times the global NMR (24/1000). According to the World Health Organization, 140 million babies are born worldwide, 90% in low-income countries and 10% in high-income countries. Approximately 99% of neonatal deaths occur in low-income countries and 1% in high-income countries; every minute, seven babies die worldwide (415 babies every hour)^3^.

In Ethiopia, infant mortality declined by 39% over the 15 years between the 2000 and 2011 EDHS, from 97 to 59 deaths per 1000 live births. Under-five mortality declined by 47% over the same period, from 166 deaths per 1000 live births to 88 deaths per 1000 live births. Neonatal mortality has also decreased from 49 deaths per 1000 live births according to EDHS 2000 to 39 deaths per 1000 live births in EDHS 2005; it remained stable at 37 deaths per 1000, as reported in EDHS 2011^8^.

The causes of neonatal death may also vary within the same country depending upon the socioeconomic status of the regional population and access to health care services. Maternal health before, during and after pregnancy. Conditions during labor, delivery and postnatal care of babies play a significant role in decreasing neonatal mortality^9^. In Ethiopia, many studies were conducted about neonatal mortality using different statistical models. In this study, the response variable is the number of death occurrences, which is limited to non-negative values. There are two problems with applying the usual linear regression model to these data. The first problem in many count data distributions is positively skewed with many observations having 0 values. The second reason is that the regression model will likely produce negative predicted values, which are theoretically impossible.

Hence, we attempted to identify the determinants of neonatal mortality in Ethiopia, focusing on socioeconomic, demographic, and environmental factors using count regression models.

Neonatal mortality is generally regarded as an important national indicator of health because it is particularly sensitive to general structural factors, like socio-economic development and basic living conditions. This study's results identified risk factors indicating the need for more intensified prenatal care and improved transport of high-risk neonates, especially in rural areas.

## Participants and methods

This cross-sectional study used about 10,641 live births born within the 5 years preceding EDHS 2016 from September to December 2015. This study is approved by the ethical review committee at Bahir Dar University, College of Science. All procedures were performed in accordance with the ethical standards of the Bahir Dar University ethics review committee declaration. No prospective studies with human participants or animals were performed by any authors for this article. Given the cross-sectional nature of this work, the need to obtain informed consent was waived for the individual participants included in the study, following the standards of the Bahir Dar University ethical review committee.

### Methods

The data source used in this study was the 2016 Ethiopia Demographic and Health Survey, conducted in Ethiopia Central Statistics Agencies (CSA) http://www.dhsprogram.com. All neonates in Ethiopia born within the 5 years preceding the survey were the source population for this study. In contrast, all neonates in Ethiopia who were in the selected enumeration areas (EAs) were considered as the study population.

A two-stage stratified cluster sampling technique was selected from a population and housing census frame for EDHS 2016. In the first stage, 645 clusters (202 in urban and 443 in rural areas) were selected with probability proportional to the cluster size and independent selection in each sampling stratum. A household listing operation was carried out in all the selected enumerations from September to December 2015. In the second stage of choice, a fixed number of 28 households per cluster were selected with an equal probability of systematic selection from the newly created household list. Finally, a nationally representative sample of 16,650 suitable households and 15,683 women was interviewed^10^. All neonatal mortality of women in Ethiopia born within the preceding 5 years of 2016 EDHS was included, while among women who did not experience neonatal mortality in the household, two EAs were not included initially in the EDHS coordinate file.

### Statistical data analysis

Different statistical techniques have been used in the analysis of collecting data. However, the method of data analysis depends on the nature of the variables included in the study and the data type of the basic variables. Depending on the above point, the study used descriptive and inferential statistics. The outcome variable of this study was neonatal mortality (number of death) and the independent factors for the outcome variable were socioeconomic variables (residence, region, source of water supply, toilet, wealth index, and mothers occupation), neonatal factors (sex of a child, birth order, a child is a twin), health care service factors (breastfeeding, place of delivery, contraceptive, number of antenatal visit during pregnancy, distance to health facility) and maternal factors (educational attainment, marital status, sex of households and mother’s age at child birth).

### Count regression models

Count regression models were developed to model data with integer outcome variables. These models can be employed to examine the occurrence and frequency of occurrence. The most popular model for count data is the Poisson model, which is based on the property that the mean and variance of the dependent variable are assumed to be equal^11^. However, this is not always the case, as the variance sometimes exceeds the mean. This is referred to as over dispersion^12^. Over-dispersion can be modeled using a negative binomial (NB) regression model, but more models accounting for over-dispersion exist. The negative binomial regression model assumes a gamma distribution for the Poisson mean with variation over the subjects^12^.

Furthermore, the response variable can be observed to show excess zero counts, contrary to what is expected, based on Poisson or negative binomial distribution. According to Ref.^13^, this implies that the count data are zero-inflated. Zero-inflated models allow for over-dispersion as well as modeling zero-inflated count data. The frequently used models for zero-inflated count data are zero-inflated Poisson (ZIP) and zero-inflated negative binomial (ZINB). This study employed count regressions to identify the determinant factors of neonatal mortality.

The ZIP model may often fail to fit such data over dispersion concerning the Poisson distribution. We extend the ZIP mixed regression model to the ZINB mixed regression model. The zero-inflated negative binomial (ZINB) regression model is an extension of the regression model. If the number of zeroes in the count distribution was excessive, then the ZIP or ZINB model more accurately fit the data than the negative binomial or Poisson model. ZINB regression is used for count data that exhibit over dispersion and excess zeros^14^. The over-dispersed data are characterized by “excess zeros”, excessively large outcomes or both. Therefore, the ZINB model accounts for “excess zeros” and extra heterogeneity in a positive outcome. The ZINB distribution is a general model for counts that nests the Poisson, NB and ZIP models^15^. The probability density function of a zero-inflated negative binomial distribution is a simple modification of the ZIP.

Suppose $yi$ is the number of neonatal deaths per household; then, the probability mass function of ZINB is given by:

$$P\left({Y}_{i}={y}_{i}\right)=\left\{\begin{array}{c}{\Pi }_{i}+\left(1-{\Pi }_{i}\right){\left(1+{\varvec{\delta}}{{\varvec{\mu}}}_{{\varvec{i}}}\right)}^{\frac{-1}{{\varvec{\delta}}}}; if y=0\\ \left(1-{\Pi }_{i}\right)\frac{\mathbf{ \Gamma }({{\varvec{y}}}_{{\varvec{i}}}+\frac{1}{{\varvec{\delta}}})({\varvec{\delta}}{{{\varvec{\mu}}}_{{\varvec{i}}}{\varvec{i}})}^{{{\varvec{y}}}_{{\varvec{i}}}}}{\mathbf{ \Gamma }\left({{\varvec{y}}}_{{\varvec{i}}}+1\right)\mathbf{ \Gamma }\left(\frac{1}{{\varvec{\delta}}}\right)}{\left(1+{\varvec{\delta}}{{\varvec{\mu}}}_{{\varvec{i}}}\right)}^{{{\varvec{y}}}_{{\varvec{i}}}}+\frac{1}{{\varvec{\delta}}}if {y}_{i}>0\end{array}\right.,\quad where; \; 0\le {\Pi }_{i}\le 1,$$

where; ${\mu }_{i}$ is the mean of the underlying negative binomial distribution, ${\Pi }_{i}$ is the logistic link function and $\delta$ is the over dispersion parameter. The ZINB distribution reduces to the ZIP distribution when the dispersion parameter $\delta$ →0. The ZINB model is a special case of a two-class finite mixture model such as the ZIP model with mean and variance^16^.

$$E({y}_{i})= (1-{\Pi }_{i}){\mu }_{i} \; \mathrm{ and },$$

$$var\left({y}_{i}\right)= \left(1-{\Pi }_{i}\right){\mu }_{i}\left(1+{\Pi }_{i}{\mu }_{i}\right).$$

The maximum likelihood parameter estimation method was used to determine the parameters that maximize the probability (likelihood) of the sample data. MLE methods are versatile and apply to most models and different data types. The generalized linear models (GLM) have no closed-form of the maximum likelihood function. To approximate the MLEs of GLM, we rely on the Newton–Raphson algorithm. The log-likelihood functions of β is:

$$\begin{aligned} l\left( {\beta ,\phi } \right) & = \sum \left\{ {\frac{{y_{i} \theta_{i} - b\left( {\theta_{i} } \right)}}{\phi } + c(y_{i} ,\phi } \right\} \\ & = \sum l_{i} (\theta_{i} ,\phi ) = \sum l_{i} , \\ \end{aligned}$$

where; $$\mathrm{\theta i}=\uptheta \left({\mathrm{X}}^{\mathrm{^{\prime}}},\upbeta \right)=\uptheta (\mathrm{\eta i})$$.

The MLEs of β are obtained by maximizing the log-likelihood functions L(β, ϕ). Where θ is a known and monotone function, the likelihood function of GLM depends on β only through the linear predictor of η.

## Results

Among the 10,641 live births in this study, approximately 364 (3.4%) neonates were died. Of the experienced neonatal mortality, 316 (3%), 36 (0.3%) and 12 (0.1%) were one, two and three children died per mother, respectively. Therefore, there are a large number of zero outcomes. However, large observations (a large number of neonatal mortality per mother) are less frequently observed. The mean number of neonatal deaths per mother was 0.04, and the variance was 0.052. Therefore, the variance in neonatal mortality is greater than the mean. This greater variance indicates the dispersion of the data (Table 1)

**Table 1:** Distribution of neonatal mortality per mother.

No of dead children in the neonatal period	Frequency	%	Number of mothers (%)
0	10,277	96.6	6855 (95.3)
1	316	3	316 (4.393)
2	36	0.3	18 (0.250)
3	12	0.1	4 (0.055)
Mean	0.04		
Variance	0.052		

Experienced neonatal mortality in urban was 40 (0.376%) from these 38 (0.357%), 2 (0.0187%) number of child mortality per mother were died at one and two neonatal periods, respectively. Regarding neonatal mortality in rural areas, 324 (3.045%) of 278 (2.612%), 34 (0.319%), and 12 (0.113%) children died per mother were at one, two and three neonatal times, respectively. Therefore, there was high neonatal mortality in rural areas compared to urban areas in one, two and three neonatal periods.

Concerning regions, Somali, Oromiya and SNNPR had the highest proportion of neonatal mortality 69 (0.648%), 50 (0.470%) and 38 (0.357%), respectively), while the Addis Ababa city administration had the lowest proportion of neonatal mortality 10 (0.094%).

In Somali region, 59 (0.554%), 4 (0.037%) and 6 (0.056), Oromiya 46 (0.432%), 4 (0.037%) and 0 (0.0%) and SNNPR 35 (0.330%), 0 (0.0%) and 3 (0.0282%) neonatal mortality were occurred at 1, 2 and 3 neonatal periods respectively (Table 2).

**Table 2:** Socioeconomic variables in the study.

Variables	Categories	Frequency (%)	No. of dead children in the neonatal period (%)				NMR per 1000
			0	1	2	3	
Region	Tigray	1033 (9.7)	1005 (9.4)	24 (0.23)	4 (0.04)	0 (0.0)	2.7
	Afar	1062 (9.9)	1027 (9.6)	28 (0.3)	4 (0.04)	3 (0.03)	32.9
	Amhara	977 (9.181)	942 (8.853)	35 (0.329)	0 (0.0)	0 (0.0)	35.8
	Oromia	1581 (14.858)	1530 (14.378)	46 (0.432)	4 (0.038)	0 (0.0)	31.6
	Somali	1505 (14.143)	1437 (13.504)	59 (0.554)	4 (0.038)	6 (0.056)	45.8
	Benishangul	879 (8.260)	843 (7.922)	28 (0.263)	8 (0.075)	0 (0.0)	40.9
	SNNPR	1277 (12.00)	1239 (11.643)	35 (0.329)	0 (0.0)	3 (0.028)	29.7
	Gambelia	714 (6.710)	693 (6.513)	19 (0.179)	2 (0.019)	0 (0.0)	29.4
	Harare	605 (5.686)	578 (5.432)	23 (0.216)	4 (0.038)	0 (0.0)	44.6
	Addis Ababa	461 (4.332)	451 (4.238)	8 (0.075)	2 (0.019)	0 (0.0)	21.7
	Dire Dawa	547 (5.140)	532 (5)	11 (0.103)	4 (0.038)	0 (0.0)	27.4
Residence	Urban	1974 (18.551)	1934 (18.175)	38 (0.357)	2 (0.019)	0 (0.0)	20.3
	Rural	8667 (81.450)	8343 (78.404)	278 (2.613)	34 (0.320)	12 (0.113)	37.4
Source of water supply	Piped water	4614 (43.36)	4474 (42.045)	123 (1.156)	14 (0.132)	3 (0.028)	30.3
	Others	6027 (56.639)	5803 (54.534)	193 (1.814)	22 (0.207)	9 (0.085)	37.2
Toilet	Use toilet	5711 (53.670)	5545 (52.109)	146 (1.372)	20 (0.188)	0 (0.0)	29
	Not use	4930 (46.33)	4732 (44.50)	170 (1.598)	16 (0.150)	12 (0.019)	40.2
Religion	Orthodox	3082 (28.963)	2984 (28.042)	85 (0.80)	10 (0.094)	3 (0.028)	31.8
	Catholic	72 (0.677)	70 (0.658)	2 (0.019)	0 (0.0)	0 (0.0)	27.77
	Protestant	1862 (17.598)	1805 (17.385)	51 (0.479)	6 (0.056)	0 (0.0)	3.1
	Muslim	5442 (51.142)	5241 (49.253)	174 (1.635)	18 (0.169)	9 (0.085)	36.9
	Others	183 (1.720)	177 (1.663)	4 (0.038)	2 (0.019)	0 (0.0)	32.8
Wealth index	Poor	6059 (56.940)	5830 (54.788)	196 (1.842)	24 (0.226)	9 (0.085)	37.8
	Middle	1693 (15.91)	1637 (15.384)	52 (0.50)	4 (0.038)	0 (0.0)	33.1
	Rich	2889 (27.150)	2810 (26.407)	68 (0.639)	8 (0.075)	3 (0.028)	27.3
Mothers occupation	Not-employed	6307 (59.3)	6092 (57.3)	184 (1.7)	22 (0.2)	9 (0.08)	34.1
	employed	4334 (40.7)	4185 (39.3)	132 (1.2)	14 (0.13)	3 (0.03)	34.4

Of the total neonatal mortality, 236 (2.218%) were male, and 128 (1.203%) were female. Of the male neonates, 207 (1.945%), 24 (0.225%), and 5 (0.047%) death were occurred at one, two and three neonatal time, respectively. In female neonates, 128 (1.203%) were 109 (1.024%), 12 (0.113%), and 7 (0.066%) children, mortality per mother, happened in one, two and three neonatal periods, respectively. Therefore, male 236 (2.218%) had greater than 128 (1.203%) female neonatal mortality (Table 3).

**Table 3:** Neonatal factors.

Variables	Frequency (%)	No. of dead children in the neonatal period (%)				NMR per 1000
		0	1	2	3	
**Sex of child**						
Male	5483 (51.5)	5247 (49.3)	207 (2.0)	24 (0.2)	5 (0.05)	43
Female	5158 (48.5)	5030 (47.3)	109 (1.0)	12 (0.1)	7 (0.1)	24.8
**Birth order**						
1	2167 (20.4)	2090 (19.6)	73 (0.7)	4 (0.04)	0 (0.0)	33.5
2–3	3338 (31.4)	3237 (30.4)	87 (0.8)	12 (0.1)	2 (0.02)	30.3
4–5	2475 (23.2)	2395 (22.5)	67 (0.6)	10 (0.1)	3 (0.03)	32.3
≥ 6	2661 (25)	2555 (24.0)	89 (0.8)	10 (0.1)	7 (0.07)	39.8
**Child is twin**						
Single	10,363 (97.4)	10,043 (94.4)	295 (2.8)	19 (0.2)	6 (0.06)	30.9
Multiple	278 (2.6)	234 (2.2)	21 (0.2)	17 (0.2)	6 (0.06)	158.3

The total number of neonatal mortality where places of delivery at home were 260 (2.443%), the public sector was 91 (0.855%), and the private sector was 13 (0.122%). From delivery at home 221 (2.077%), 27 (0.254%) and 12 (0.113%), at public sector 84 (0.789%), 7 (0.066%) and 0 (0.0%), and private sector also 11 (0.103%), 2 (0.019%) and 0 (0.0%) number of children per mothers died one, two and three neonatal period respectively. Therefore, delivery at home has the highest neonatal mortality compared to the private and public sectors of delivery.

The total number of neonatal deaths not experiencing breastfeeding was 221 (2.077%), and 143 (1.34%) were experiencing breastfeeding. From not experiencing breastfeeding 195 (1.832%), 20 (0.210%), and 6 (0.056%) and experiencing breastfeeding 121 (1.137%), 16 (0.150%), and 6 (0.056%) children died in one, two and three neonatal periods respectively. Therefore, 221 (2.077%) women who did not experience breastfeeding had greater neonatal mortality than 143 (1.344%) women who experienced breastfeeding. From our study findings, we had observed a difference between facilities-based birth and home-based birth in terms of neonatal mortality, which indicates about 221 (2.1%) death has occurred in home-based delivery services. In comparison, 95 (0.9%) of death occurred in facilities-based birth delivery in the first neonatal period. This difference implies neonatal mortality was higher in home-based birth delivery compared to facilities-based birth delivery in all neonatal periods (Table 4).

**Table 4:** Health care service factors.

Variables	Frequency (%)	No of died children in the neonatal period (%)				NMR per 1000
		0	1	2	3	
**Breast feeding**						
No	3821 (35.9)	3600 (33.8)	195 (1.8)	20 (0.2)	60 (0.1)	57.8
Yes	6820 (64.1)	6677 (62.7)	121 (1.1)	16 (0.1)	6 (0.1)	20.9
**Place of delivery**						
Home	7155 (67.2)	6895 (64.8)	221 (2.1)	27 (0.3)	12 (0.1)	36.3
Public sector	3023 (28.4)	2932 (27.6)	84 (0.8)	7 (0.1)	0 (0.0)	30.1
Private sector	463 (4.4)	450 (4.2)	11 (0.1)	2 (0.02)	0 (0.0)	28
**Contraceptive**						
Not using	7888 (74.1)	7607 (71.5)	245 (2.3)	24 (0.23)	12 (0.1)	35.6
Using	2753 (25.9)	2670 (25.1)	71 (0.7)	12 (0.1)	0 (0.0)	30.1
**No antenatal visit during pregnancy**						
No visit	3981 (37.4)	3809 (35.8)	150 (1.4)	16 (0.2)	6 (0.1)	43.2
1–4 visit	4676 (43.9)	4536 (42.6)	123 (1.2)	13 (0.1)	4 (0.04)	29.9
5–10 visit	1924 (18.1)	1873 (17.6)	42 (0.4)	7 (0.1)	2 (0.04)	26.5
11–15 visit	44 (0.4)	43 (0.4)	1 (0.01)	0 (0.0)	0 (0.0)	22.7
≥ 16 visit	16 (0.2)	16 (0.15)	0 (0.0)	0 (0.0)	0 (0.0)	–
**Distance to health facility**						
Big problem	5834 (54.8)	5619 (96.3)	179 (3.1)	24 (0.4)	12 (0.2)	36.9
Not big problem	4807 (45.2)	4658 (45.3)	137 (2.9)	12 (0.2)	0 (0.0)	30.9

Of the total number of neonatal deaths for uneducated mothers, 6838 (64.261%) were primary, 2678 (25.167%) were secondary, and more than 1,125 (10.572%). Child mortality was about 218 (2.049%), 28 (0.263%), 9 (0.085%) among uneducated mothers in one, two and three neonatal periods, respectively. Similarly, among primary education mothers, there were about 73 (0.686%), 4 (0.037%) and 3 (0.028%) death of neonatal at one, two and three neonatal period, respectively, and among mothers whose education were secondary and above 25 (0.235%), 4 (0.037%) and 0 (0.0%) number of died children per mother occurred at one, two and three neonatal time respectively. There was a decreasing trend in neonatal mortality concerning the mother's educational level (Table 5).

**Table 5:** Demographic (maternal) factors.

Variables	Frequency (%)	No of dead children in the neonatal period (%)				NMR per 1000
		0	1	2	3	
**Educational attainment**						
No education	6838 (64.3)	6583 (61.9)	218 (2.0)	28 (0.3)	9 (0.1)	37.3
Primary	2678 (25.2)	2598 (24.4)	73 (0.7)	4 (0.04)	30 (0.03)	29.9
Sec. and above	1125 (10.6)	1096 (10.3)	25 (0.2)	4 (0.04)	0 (0.0)	32.6
**Marital status**						
Married	9903 (93.1)	9564 (89.9)	291 (2.7)	36 (0.34)	12 (0.1)	34.2
Widowed	135 (1.3)	129 (1.2)	6 (0.06)	0 ( (0.0)	0 (0.0)	44.4
Divorced	328 (3.1)	318 (3)	10 (0.09)	0 (0.0)	0 (0.0)	30.5
Other	275 (2.6)	266 (2.5)	9 (0.08)	0 (0.0)	0 (0.0)	32.7
**Sex of HH**						
Male	8383 (78.8)	8077 (75.9)	264 (2.5)	30 (0.3)	12 (0.1)	36.5
Female	2258 (21.2)	2200 (20.7)	52 (0.5)	6 (0.06)	0 (0.0)	25.7
**Mother’s age at child birth**						
15–19	404 (3.8)	390 (3.7)	14 (0.1)	0 (0.0)	0 (0.0)	34.7
20–24	2171 (20.4)	2088 (19.6)	72 (0.7)	8 (0.08)	3 (0.03)	38.2
25–29	3161 (29.7)	3064 (28.8)	80 (0.8)	14 (0.1)	3 (0.03)	30.7
30–34	2360 (22.2)	2295 (21.6)	61 (0.6)	4 (0.04)	0 (0.0)	27.5
35–39	1697 (15.9)	1628 (15.3)	57 (0.5)	6 (0.06)	60 (0.06)	40.7
40–44	647 (6.1)	623 (5.8)	20 (0.2)	4 (0.04)	0 (0.0)	37.1
45–49	201 (1.9)	189 (1.8)	12 (0.1)	0 (0.0)	0 (0.0)	59.7

The model selection methods include information criteria AIC, BIC and log-likelihood values. The models are described to fit the data. AIC, BIC and the corresponding log-likelihood value of each model were presented in Table 6. The Poisson, negative binomial, zero-inflated Poisson regression models had the largest value of AIC and BIC, demonstrating a poor fit to the data. The remaining ZINB model had smaller AIC, BIC and the largest log-likelihood value. Therefore, ZINB models are appropriate and preferred for the number of Neonatal Mortality per mother.

**Table 6:** Model selection criteria for the count regression model.

Model	Poison	NB	ZIP	ZINB
AIC	3392.8	3328.6	3328.7	3320.4
BIC	3683.7	3626.8	3619.5	3371.8
Log-likelihood	− 1609.9	− 1576.8	− 1532.9	− 1529.7

According to the result of Table 7, in ZINB model, the variables breastfeeding, child twin, number of antenatal visits during pregnancy, the number of birth order, place of residence, sex of household head and region are statistically significant factors of neonatal mortality per mother in Ethiopia.

**Table 7:** Parameter estimates for the ZINB model for the count data.

Parameter	Estimate	SE (95% CI)	IRR
Intercept	− 1.11***	1.03 (− 3.14, − 0.91)	0.33
**No. AN. visit pregnancy (No antenatal visit, ref)**			
1–4 times visit	− 0.72***	0.22 (− 1.15, − 0.29)	0.48
5–10 times visit	− 0.33***	0.40 (− 1.11, − 0.46)	0.72
11–15 times visit	− 0.86	1.89 (− 4.57, 2.85)	0.42
16 and above visit	− 8.23	31.1 (− 69.19, 52.73)	0.003
**No. birth order (≥ 6, ref)**			
1	− 0.97***	0.56 (− 2.06, − 0.13)	0.38
2–3	− 0.73***	0.35 (− 1.42, − 0.04)	0.48
4–5	− 0.53	0.33 (− 1.17, 0.12)	0.59
**Sex of child (male, ref)**			
Female	− 0.08	0.22 (− 0.51, 0.35)	0.92
**Contraceptive (using, ref)**			
Not using	0.34	0.25 (− 0.15, 0.83)	1.40
**Edu. (sec. and above, ref)**			
No education	− 0.23	0.49 (− 1.19, 0.72)	0.79
Primary	− 0.06	0.46 (− 0.96, 0.84)	0.94
**Mothers age at birth (45–49, ref)**			
15–19	0.66	1.09 (− 1.48, 2.79)	1.93
20–24	− 0.06	0.7 (− 1.42, 1.3)	0.94
25–29	0.09	0.67 (− 1.21, 1.4)	1.097
30–34	− 1.14	0.62 (− 2.36, 0.08)	0.32
35–39	− 1.16	0.62 (− 2.37, 0.06)	0.32
40–44	− 1.04	0.67 (− 2.36, 0.28)	0.35
**Residence (urban, ref)**			
Rural	1.38***	0.57 (0.26, 2.5)	3.99
**Region (Tigray, ref)**			
Addis Ababa	0.70	0.9 (− 1.04, 2.45)	2.02
Afar	0.51	0.61 (− 0.68, 1.7)	1.7
Amhara	0.43	0.47 (− 0.49, 1.35)	1.53
Benishangul	0.58	0.57 (− 0.53, 1.69)	1.78
Dire Dawa	1.13***	0.59 (0.03, 2.28)	3.085
Gambela	0.18	0.54 (− 0.88, 1.23)	1.19
Harare	0.93***	0.47 (0.001, 1.8588)	2.53
Oromia	0.24	0.51 (− 0.76, 1.2475)	1.27
SNNPR	− 0.04	0.45 (− 0.92, 0.84)	0.96
Somali	0.37	0.48 (− 0.58, 1.32)	1.45
**Sex of HH (male, ref)**			
Female	− 0.42***	0.32 (0.21, 1.04)	0.66
**Water (piped water, ref)**			
Others	0.019	0.24 (− 0.45, 0.49)	1.02
**Toilet (toilet use, ref)**			
No toilet uses	− 0.05	0.23 (− 0.50, 0.41)	0.95
**Wealth index (rich, ref)**			
Middle	− 0.45	0.33 (− 1.1, 0.2)	0.64
Poor	− 0.14	0.33 (− 0.79, 0.51)	0.87
**Breastfeeding (no, ref)**			
Yes	− 0.98***	0.26 (− 1.49, − 0.47)	0.38
**Child twin (single, ref)**			
Multiple	2.14***	0.26 (1.63, 2.65)	8.53
**Distance to health facilities (not big problem = ref)**			
Big problem	− 0.002	0.22 (− 0.43, 0.43)	0.99
**Mother’s occupation (not employed = ref)**			
Employed	− 0.24	0.26 (− 0.75, 0.3)	0.78
**Place of delivery (public sector = ref)**			
Private sector	− 0.51	0.41 (− 1.3, 0.3)	0.6
Private sector	0.60	0.71 (− 0.79, 2.0)	1.83

The average number of neonatal deaths experienced during breastfeeding was lower (IRR = 0.38) than that experienced by mothers who did not experience breastfeeding their child. The average neonatal mortality difference in rural residences was increased by IRR = 3.99 times compared to the urban residence of mothers. The average neonatal mortality difference in multiple births was increased by IRR = 8.53 times compared with a single birth. The average neonatal mortality differences at antenatal visits (1–4) and (5–10) during pregnancy were decreased by IRR = 0.48 and IRR = 0.72, respectively, compared to those at no antenatal visits during pregnancy. The average neonatal mortality difference in the female household head was decreased by IRR = 0.66 times compared to that in the male household head. The average number of neonatal mortality differences in the first, second up to the third number of birth order has decreased by (IRR = 0.38, IRR = 0.48) times, respectively, as compared to six and above the number of birth order. The average neonatal mortality difference in Dire Dawa and Harari increased by IRR = 3.085 and IRR = 2.53, respectively, compared to that in the Tigray region (Table 7).

The number of neonatal mortalities in the zero-inflated model was modeled by including the covariates antenatal visits during pregnancy, breastfeeding, sex of a child, distance to health facilities and mother’s occupation, which had statistically significant effects on the probability of being in the zero number of neonatal mortalities. The estimated average number of neonatal mortalities that became zero when experiencing breastfeeding was lower by IRR = 0.045 than when not experiencing breastfeeding, while the other variables remained constant. The average number of neonatal deaths becomes zero when having a female child is lower by IRR = 0.49 compared to a male child, keeping other variables constant. The average number of neonatal deaths becomes zero, having (1–4) fewer antenatal visits during pregnancy (IRR = 0.42) than no antenatal visit during pregnancy, keeping other variables constant. The average number of neonatal deaths becomes zero, with a large problem higher by IRR = 0.8 compared to a non-large problem to reach health facilities, keeping other variables constant. The average number of neonatal deaths becomes zero when having employed mothers is higher by IRR = 1.68 as compared to not employing mothers, keeping other variables constant (Table 8).

**Table 8:** Zero inflation parameter estimates.

Parameter	Estimate	SE	Wald 95% C. I		IRR
Intercept	3.81***	1.7	0.50	7.1	45
**No. AN. visit pregnancy (No antenatal visit, ref)**					
1–4 times visit	− 0.85***	0.4	− 1.6	− 0.11	0.42
5–10 times visit	− 0.24	0.67	− 1.55	1.07	0.79
11–15 times visit	− 1.49	2.6	− 6.56	3.59	0.22
16 and above visit	− 4.7	0.000	− 4.70	− 4.70	0.009
**No. birth order (≥ 6, ref)**					
1	− 1.61	0.96	− 3.5	0.28	0.20
2–3	− 0.93	0.61	− 2.13	0.27	0.39
4–5	− 0.79	0.60	− 1.97	0.39	0.45
**Sex of child (male, ref)**					
Female	− 0.70***	0.34	0.025	1.38	0.49
**Contraceptive (using, ref)**					
Not using	0.62	0.43	− 0.22	1.47	1.86
**Edu. (sec. and above, ref)**					
No education	− 0.71	0.81	− 2.3	0.87	0.49
Primary	− 0.19	0.73	− 1.63	1.25	0.83
**Mothers age at birth (45–49, ref)**					
15–19	1.014	1.61	− 2.14	4.17	2.76
20–24	− 0.3	1.077	− 2.41	1.81	0.74
25–29	0.34	0.99	− 1.6	2.28	1.41
30–34	− 1.33	0.96	− 3.21	0.54	0.26
35–39	− 1.86	0.97	− 3.76	0.028	0.16
40–44	− 1.17	1.04	− 3.21	0.87	0.31
**Residence (urban, ref)**					
Rural	1.33	1.08	− 0.8	3.46	3.79
**Region (Tigray, ref)**					
Addis Adaba	0.37	1.74	− 3.03	3.77	1.45
Afar	0.85	1.04	− 1.18	2.88	2.34
Amhara	0.31	0.75	− 1.16	1.78	1.37
Benishangul	0.07	0.91	− 1.72	1.85	1.07
Dire Dawa	1.28	0.93	− 0.55	3.11	3.60
Gambela	0.28	0.87	− 1.43	1.99	1.32
Harare	0.26	0.80	− 1.32	1.84	1.3
Oromia	0.36	0.85	− 1.31	2.03	1.43
SNNPR	− 0.34	0.76	− 1.83	1.15	0.71
Somali	− 0.27	0.82	− 1.87	1.32	0.76
**Sex of HH (male, ref)**					
Female	− 0.11	0.55	− 1.18	0.96	0.9
**Water (piped water, ref)**					
Others	0.097	0.4	− 0.69	0.88	1.102
**Toilet (toilet use, ref)**					
No toilet uses	− 0.44	0.37	− 1.18	0.29	0.64
**Wealth index (rich, ref)**					
Middle	− 1.07	0.58	− 2.20	0.06	0.34
Poor	− 0.56	0.54	− 1.62	0.50	0.57
**Breastfeeding (No, ref)**					
Yes	3.09***	0.45	− 3.98	− 2.21	0.045
**Child twin (single, ref)**					
Multiple	0.11	0.45	− 0.87	1.08	1.11
**Distance to health facilities (not big problem = ref)**					
Big problem	− 0.22***	0.36	− 0.92	− 0.48	0.80
**Mother’s occupation (not employed = ref)**					
Employed	0.52***	0.44	− 1.39	− 0.34	1.68
**Place of delivery (public sector = ref)**					
Home	− 0.66	0.65	− 1.94	0.61	0.51
Private sector	0.78	1.01	− 1.2	2.76	2.18

## Discussion

Neonatal mortality erodes the potential economic labor force, thus plunging the country into a human resource disaster and retarding development. Children are the human resource bank of every nation and nationality of the world. However, they are more exposed to diseases and other health risks. Therefore, this study attempted to identify the socioeconomic, demographic, health care service and environmental-related factors of neonatal mortality based on EDHS 2016 data.

From the result of this study, the prevalence of neonatal mortality was about 364 (3.4%), which was in line with the study conducted in Ethiopia^17^. But this study's result is lower than the result shown in Ethiopia^18^. The possible reason for the difference in the prevalence was due to the difference in the sample size included in the study, the study period difference and also the statistical methodology we applied.

The results of this study indicated that breastfeeding, child twin, number of antenatal visits during pregnancy, number of birth orders, place of residence, sex of household head, sex of a child, distance to health facilities, mother’s occupation and region had a statistically significant influence on the number of neonatal deaths in Ethiopia. The risk of neonatal mortality associated with multiple births is very high relative to single births, and this study is consistent with other studies^1^. The risk of neonatal mortality associated with rural residence is very high relative to an urban residence, and this study is consistent with a previous study^17^. The reason might be that mothers living in rural areas have limited access to health facilities, maternal healthcare services, and health care professionals for neonatal mortality in rural areas^1^.

The study result showed that; experienced breastfeeding children have a lower risk of neonatal mortality than children who do not experience breastfeeding. This finding seemed to be consistent with other studies^19^. The possible reason might be that the harmful effects of infections related to neonatal deaths can be prevented by breast milk, especially when practicing breast milk feeding within the first 1 h after birth. This practice minimizes the risk of death and helps wealth and brain development^20^. Additionally, increasing the number of antenatal visits during pregnancy reduces the risk of neonatal mortality, and this finding seemed to be consistent with other studies^1^. This might be due to the lack of access to health care, especially basic and emergency obstetric care, related to neonatal mortality in Ethiopia^21^.

In this study, rural neonatal were experienced higher mortality than their urban counterparts. The main reason might be a low maternal health service utilization level amongst rural women compared with their urban counterparts. In addition, health institutions are typically concentrated in urban areas and women in urban tended to benefit from better awareness and access to services than rural mothers. Moreover, previous studies have found that rural women are more readily influenced by traditional practices contrary to modern health care. These elements potentially explain the close connection between residence and neonatal mortality. This result was similar to the study result of Refs.^22,23^.

The risk of neonatal mortality associated with the Dire Dawa and Hareri regions is very high relative to the Tigray region, and this study is consistent with other studies^17^. This might be due to the difference in the health facility among regions of the country.

The study also showed that children born from employed mothers have a higher mortality risk than non-employed mothers. This finding is consistent with previous studies^24,25^. The difference could be attributed to the reasons that most employed mothers were engaged in the informal sector like agriculture and sales, which this study has further confirmed to be risky for children's survival. And also, this negative relationship of mother employment and neonatal mortality could be due to the non-availability of adequate time for childcare among working mothers.

Furthermore, birth order has been associated with a variety of adverse life outcomes for children, including morbidity and mortality. This study result found that short preceding birth intervals (first, second to third birth orders) have higher odds of neonatal mortality, a finding that is consistent with most previous findings. This study is consistent with an earlier study^26^. However, in contrast to previous findings, our analyses found that long preceding birth intervals have lower odds of mortality. The possible argument for the impact of birth order on health and mortality risks has been formulated within the biological depletion hypothesis. According to this hypothesis, later-born children are more likely than their older siblings to be less healthy because they are born to older mothers who have already given birth to a number of children before and therefore are physiologically less able to produce stronger children. However, there is some evidence to the contrary that has found t older siblings, particularly first-borns, are at greater risk of diseases and death because they are typically born to younger women who are neither mature and experienced enough in mothering nor economically well enough off to provide adequate emotional and material resources to their newborn babies^27^.

The risk of neonatal mortality associated with female children is lower than that associated with male children, and this study is consistent with a previous study^28^. The study conducted in India and Pakistan also confirmed that neonatal mortality risks were higher among male infants than their female counterparts^29^. This may be due to biological differences between male and female neonates. That is, female neonates had a stronger immune system than male neonates’ due to genetic differences between females and males. Newborn girls have a biological advantage in survival over newborn boys^27,30^.

Moreover, the study revealed that children born from the big problem of distance to a health facility have a higher risk of neonatal mortality than those not with big problems with reaching a health facility. This study is consistent with the previous study^21^. The possible reasons might be lack of ambulance or transportation problems due to low safety of roads which turn results in a delay of the ambulance to referrals hospitals or clinics, most especially for remote areas.

### Limitation of the study

All research has its limitation, and it is very important to present the limitation of our study to our audience (other researchers, journal editors and article reviewers).

Therefore, the limitations of this study are that the data are from secondary sources and cannot be centrally extrapolated between neonatal death and their determinants, nor can they explain geographical variation. Future studies need to address spatial effects to explain the fluctuations in neonatal death and prevalence caused by geographic differences.

## Conclusion

The main objective of this study was to identify the determinant factor of neonatal mortality in Ethiopia based on EDHS 2016 data with the application of a spatial and count regression model. The study was based on secondary data obtained from the central statistical agency of Ethiopia. For this study, the ZINB regression model was the most appropriate. This result suggested that the ZINB model is most suitable to deal with the problem of over dispersion and excess zero values simultaneously.

The prevalence of neonatal mortality in Ethiopia was higher (3.4%). This study also investigated high-risk areas of neonatal mortality in Somali, Afar, border of Tigray and afar, border of Amhara and Afar, Oromia, border of Amhara and Oromiya, and Hararie and Dire Dawa regions of the country. In this study, the risk factors for neonatal mortality were multiple birth types, rural residences, health facilities, environmental places of Dire Dawa administrative city and Hararie Region, and employed mothers as significant factors of neonatal mortality that increased the risk of death during the neonatal period. However, having early breastfeeding, a female household head, antenatal visits (1–4) and (5–10) during pregnancy and having the first and two to three birth orders decreased the risk of neonatal death.

Based on the finding of this study, we strongly recommend that attention be paid to socio-economic, demographic, environmental, and maternal health service factors to ensure a significant reduction in neonatal mortality. The Federal Ministry of Health needs to prioritize the high risk of neonatal death and improve early maternal and child health care. Efforts are underway to expand educational programs to educate mothers about the benefits of breastfeeding, prenatal screening, birth order, mother's profession, and out-of-hospital residences to reduce neonatal mortality.

## Abbreviations

AIC: Akaki information criterion
ANC: Antenatal care
BIC: Bayesian information criteria
DHS: Demographic health survey
EDHS: Ethiopian demographic health survey
IRR: Incidence rate ratio
LR: Likelihood ratio
MLE: Maximum likelihood estimation
NB: Negative binomial
NBR: Negative binomial regression model
NM: Neonatal mortality
NMR: Neonatal mortality rate
ZIP: Zero-inflated poisson
ZINB: Zero-inflated negative binomial
